# Freeze‐Derived Microporous Biomaterials for Tissue Engineering Applications

**DOI:** 10.1002/smmd.70035

**Published:** 2026-05-21

**Authors:** Shuangshuang Miao, Xingkui Guo, Chenhui Bai, Wei Zhou, Yiwen Wu, Wanchuan Ding, Jingjing Gan

**Affiliations:** ^1^ Department of Rheumatology and Immunology Nanjing Drum Tower Hospital School of Energy and Environment Southeast University Nanjing China; ^2^ Department of Mechanical Engineering National University of Singapore Singapore Singapore; ^3^ Department of Civil and Environmental Engineering Imperial College London London UK

**Keywords:** biomedical, freeze‐casting, ice‐template, scaffolds, tissue engineering

## Abstract

Tissue engineering holds immense promise to revolutionize regenerative medicine by enabling the fabrication of functional, patient‐specific tissues and organs for clinical translation, yet it continues to face persistent challenges in designing scaffolds that simultaneously recapitulate native tissue architecture, support cell viability, and enable efficient mass transport. Traditional fabrication has moved the field forward, yet routinely falls short of producing hierarchical, anisotropic, biomimetic structures under gentle conditions. Ice‐templating (or freeze‐casting), which is based on freeze‐induced microphase separation, reframes the problem as crystal‐growth engineering. This review summarizes current fabrication strategies and their underlying mechanism of ice‐templating technology from physical and chemical perspectives. We then highlight recent advances in ice‐templating for tissue engineering application fields such as 3D cell culture, wound healing, bone regeneration, nerve repair, and liver support, emphasizing the relationship between microstructure and biomedical functional performance. Finally, we discuss the key challenges in translating ice‐templated biomaterials from laboratory research to clinical practice and outline future directions to fully harness this versatile biomedical strategy.

## Highlights

- Crystal‐growth–guided ice‐templating strategies enabling hierarchical and anisotropic biomaterials for tissue engineering are first comprehensively summarized from physical and chemical perspectives.
- Recent progress in ice‐templated biomaterials for tissue engineering, including 3D cell culture, wound healing, bone regeneration, nerve repair, and liver support, is systematically reviewed with emphasis on structure–function relationships.
- Current challenges and future opportunities for translating ice‐templated biomaterials from laboratory studies to clinical applications are discussed.

## Graphical abstract

Ice‐templating demonstrates significant potential to revolutionize tissue engineering through freeze‐induced microphase separation for fabricating hierarchical, biomimetic scaffolds. This review comprehensively examines recent advances in mechanisms and applications in tissue engineering, such as 3D cell culture, wound healing, bone regeneration, nerve repair, and liver support, aiming to bridge design challenges and guide clinical translation.

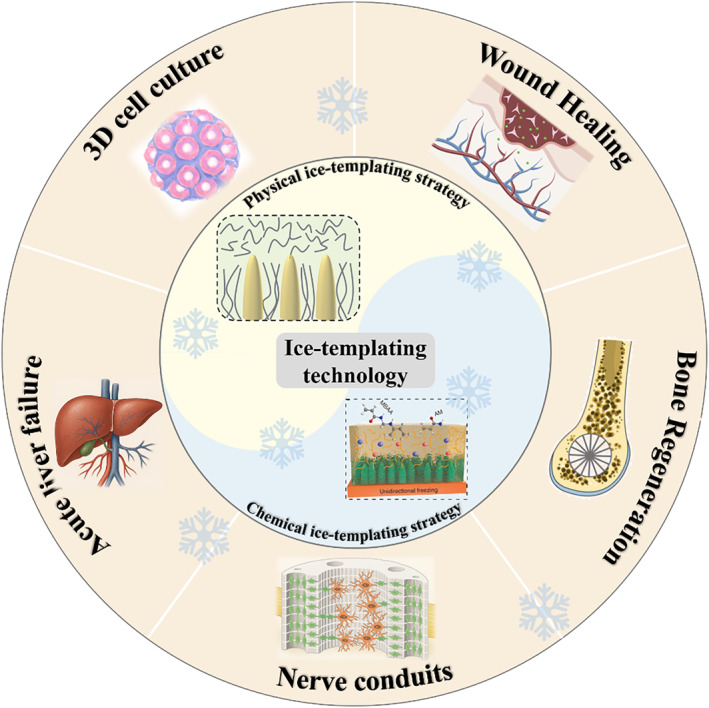

## 1 Introduction

Tissue engineering has emerged as a key driver of regenerative medicine, aiming to generate functional, patient‐customized tissues and organs that can be translated into clinical practice [1, 2, 3, 4, 5, 6]. Over the past decades, remarkable progress has been made in stem cell biology, biomaterials science, and bioprinting technologies, all of which have contributed to the vision of constructing living substitutes that can restore or replace damaged tissues [7]. Nevertheless, the successful translation of tissue engineering concepts into clinical reality remains hindered by persistent challenges. The key challenge is to design scaffolds that not only provide mechanical support but also faithfully recapitulate the hierarchical architecture of native tissues [3]. Such scaffolds must present pores of appropriate size and orientation to orchestrate cell adhesion, proliferation, and differentiation, while simultaneously maintaining high levels of bioactivity and biocompatibility. Furthermore, efficient nutrient and oxygen transport through interconnected porous networks is indispensable for sustaining cell viability and functional integration with host tissues [8]. Achieving this delicate balance between structural fidelity, biological performance, and physiological transport remains a formidable obstacle, underscoring the urgent need for innovative fabrication strategies capable of constructing biomimetic architectures.

Freeze‐casting has emerged as a powerful technique for producing highly controlled porous architectures [9, 10, 11, 12]. The process relies on the directional growth of ice crystals as a removable template: during freezing, the solidifying solvent (typically water) expels suspended or dissolved materials into the voids among the developing ice branches [13, 14]. Subsequent removal of the ice template, traditionally by freeze‐drying, results in a porous replica whose structure directly reflects the morphology, size, and orientation of the ice crystals [15, 16]. By adjusting parameters such as freezing temperature, cooling rate, additives, and solid content, ice‐templating can yield a broad range of pore geometries, from isotropic cellular pores to highly aligned lamellar or columnar channels [17, 18]. The technique is applicable to a wide variety of materials [19, 20], including ceramics [21], polymers [22], hydrogels [23, 24], and composites [25], and is conducted under relatively mild conditions, making it particularly suitable for processing biomolecules and bioactive agents. Although several reviews on ice‐templating have been published [26, 27, 28], they mainly focus on structural mechanics [29, 30], energy applications [31, 32], environmental applications [33, 34], and electromagnetic applications [35]. In contrast, the biomedical applications of ice‐templated materials remain insufficiently explored and lack a systematic summary, particularly with respect to providing methodological guidance for the design of ice‐templated porous structures tailored to biomedical needs.

Owing to its unique capability to construct hierarchical, anisotropic, and biomimetic structures, ice‐templating has gained increasing attention for tissue engineering applications [36, 37]. Recent studies have demonstrated its effectiveness in fabricating hydrogel matrices with interconnected micropores for enhanced cell infiltration and vascularization and bone scaffolds with aligned lamellar channels to guide osteogenesis [38]. In drug delivery, ice‐templated porous hydrogels and aerogels enable controlled drug release through tailored diffusion pathways [39, 40], while in biosensing, conductive ice‐templated hydrogels provide high surface area and efficient electron transport [41, 42]. These successes underscore the potential of ice‐templating as a structural design platform for biomedical materials with optimized biological and functional performance. Despite these advances, several challenges remain. The relationships between ice crystal growth dynamics, resulting pore architecture, and the subsequent biomedical responses are not yet fully elucidated, limiting the ability to rationally design materials for specific biomedical functions [43]. The integration of ice‐templating with emerging technologies, such as additive manufacturing, controllable freezing fields, nanomaterial incorporation, and bioinspired or AI‐assisted structural design, offers exciting opportunities to expand its capabilities [44].

**FIGURE 1:**
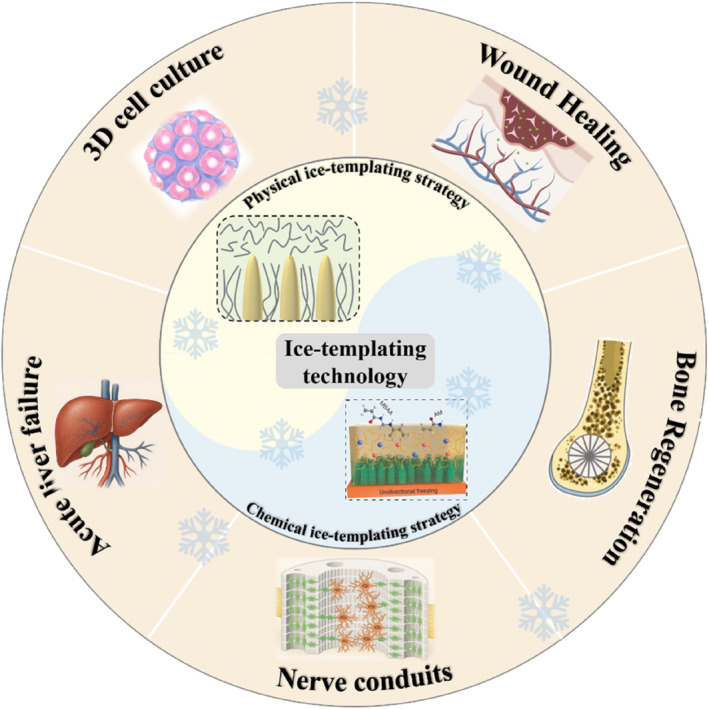
Overview of freeze‐derived porous biomaterials for tissue engineering applications.

This review aims to provide a comprehensive overview of recent developments and challenges in ice‐templating technology for tissue engineering applications (Figure 1). We focus on fabrication strategies and mechanisms (physical and chemical), structural control methods (pore size, interconnectivity, anisotropy, hierarchy), and microstructure–function relationships across cell culture, wound healing, bone regeneration, nerve conduits, and liver support. We also identify key barriers to clinical translation, including mechanistic mapping, materials trade‐offs, manufacturing reproducibility, and standardization, and outline future directions to operationalize ice‐templating as a predictable, scalable platform for tissue engineering applications.

## 2 Ice‐Templating Fabrication Technology and Its Underling Mechanism

Since the first *Science* paper on freeze‐casting was published in 2006 [45], the technology has undergone nearly 2 decades of development and has been applied across a wide range of fields, including biomimetic structural mechanical application [46, 47, 48], thermal insulation application [49], energy field [50, 51, 52], environmental application [53, 54], electrical application, etc. [5, 55, 56, 57]. The technical feedback from these diverse applications has, in turn, continuously driven the evolution of freeze‐casting itself. Numerous other techniques have been integrated with freeze‐casting to treat precursor solutions [58]. For instance, thermal engineering approaches, such as using finned or wedge‐shaped substrates to generate temperature gradients, have given rise to bidirectional ice‐templating technology [59]. The flexibility and precision of 3D printing in designing macroscopic architectures have been combined with the microscale structural control of freeze‐casting to develop 3D‐freeze‐printing technology for fabricating multiscale functional devices [60, 61]. In chemistry, free radical polymerization can be initiated under frozen conditions, enabling in situ polymerization of ice‐templated materials without the need for subsequent freeze‐drying [62], thereby significantly reducing both time and economic costs. Many other such examples exist [63]. Therefore, this chapter reviews the current advancements in freeze‐casting technology and categorizes them into physical and chemical approaches (refer to Table 1 for details), aiming to inspire scientists and engineers across disciplines to address technical challenges in their respective fields using the ice‐based technology [6, 64].

**TABLE 1:** Comparison between physical and chemical ice‐templating approaches.

	Physical ice‐templating (freeze‐drying)	Chemical ice‐templating (physicochemical synergistic approach)
Core principle	Purely physical process: Relies on ice crystal growth to exclude solutes, followed by sublimation to remove the ice template, forming a porous structure.	Physical shaping + chemical fixation: Utilizes ice growth for physical confinement/templating while introducing in situ chemical reactions to solidify the structure before ice melting.
Formation/Fixation mechanism	Structure is maintained by the dried solid skeleton after sublimation. Highly susceptible to structural collapse or shrinkage during drying or rehydration due to capillary forces.	Structure is locked in situ through reactions (polymerization, crosslinking, complexation) while the ice exists or at the moment of melting. Significantly enhances structural stability and prevents collapse.
Process efficiency	Very low. The sublimation drying process is extremely slow (often taking days), forming a process bottleneck.	High. Chemical reactions (especially photopolymerization) or ice‐dissolution‐complexation are very rapid (seconds to minutes), drastically reducing total fabrication time.
Structural fidelity	Relatively low. Drying shrinkage and ice melting can cause pore deformation, closure, or overall shrinkage.	High. Chemical reactions “cast” and solidify the ice‐template before its removal, highly preserving the intricate architecture imparted by the ice, including interconnectivity and anisotropy.
Functional integration	Weak. Primarily provides physical structure. Functionalization relies on precursor modification or post‐processing.	Strong. Functional components (conductive polymers, nanofillers, responsive monomers) can be directly incorporated during fabrication, enabling integrated structure‐function manufacturing.
Processable forms	Mainly bulk porous scaffolds or membranes.	More diverse forms, including high‐strength hydrogels, flexible films, complex structural‐color devices, high‐performance separation membranes, etc.
Key advantages	Simple principle, broad material compatibility, capable of producing scalable porous aligned structures.	High efficiency, high fidelity, multifunctionality. Solves key pain points of traditional methods: Low efficiency and structural instability.
Key limitations	Time‐ and energy‐consuming, structure prone to collapse, limited functionality, difficult to rehydrate without shape loss.	May involve chemical reagents (monomers, initiators) requiring biocompatibility consideration; process control is more complex.

### 2.1 Physical Ice‐Templating Strategy

#### 2.1.1 Isotropic Ice‐Templating Strategy

**FIGURE 2:**
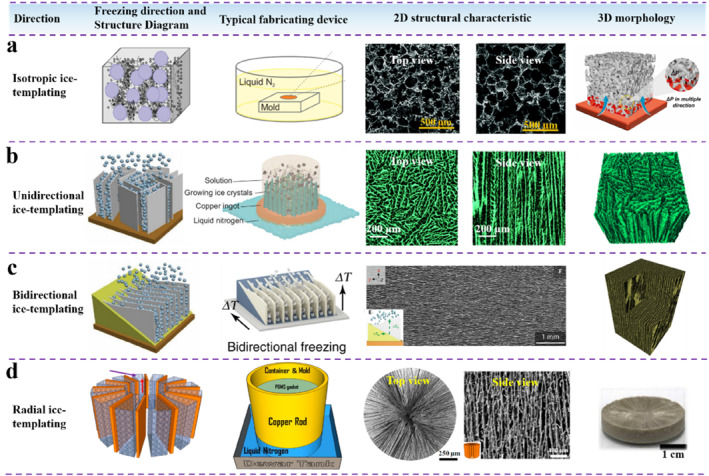
Common physical ice‐templating strategy. (a) Isotropic ice‐templating strategy. Freezing direction and structure diagram [67], typical fabricating device [66], 2D structural characteristic [69], 3D morphology [70]. (b) Unidirectional ice‐templating strategy. Freezing direction and structure diagram and typical fabricating device [80], 2D structural characteristic and 3D morphology [82]. (c) Bidirectional ice‐templating strategy. Typical fabricating device [92], freezing direction and structure diagram, 2D structural characteristic and 3D morphology [59]. (d) Radial ice‐templating strategy [97]. (a) Adapted with permission [67]. Copyright 2017, Wiley‐VCH. Adapted with permission [66]. Copyright 2018, American Chemical Society. Adapted with permission [69]. Copyright 2016, Royal Society of Chemistry. Adapted with permission [70]. Copyright 2022, Elsevier. (b) Adapted under terms of the CC‐BY license [80]. Copyright 2018, The Authors, published by Oxford University Press. Adapted with permission [82]. Copyright 2018, The Authors, published by AAAS. (c) Adapted under terms of the CC‐BY license [92]. Copyright 2019, The Authors, published by Springer Nature. Adapted with permission [59]. Copyright 2015, The Authors, published by AAAS. (d) Adapted with permission [97]. Copyright 2018, American Chemical Society.

During freeze‐casting, the aqueous phase in the precursor solution nucleates and crystallizes, while the non‐aqueous components are expelled by the growing ice crystals, leading to microphase separation [36, 65]. It is evident that freeze‐casting relies on the drilling pore effect of ice crystal growth to ultimately produce a porous structure. Therefore, controlling the direction of freezing enables tailoring of pore alignment. If the precursor is directly immersed in liquid nitrogen [66], refrigerator, or a low‐temperature alcohol solution (e.g., −80°C) [67, 68], random freezing occurs, and ice crystals grow freely in all directions (Figure 2a). After subsequent freeze‐drying, isotropic ice‐templated materials with random pores distribution are obtained [69, 70]. In general, the isotropic ice‐templating strategy focuses less on pore orientation and more on pore size. Thus, the freezing–assembly process is typically regulated by adjusting the freezing temperature and the type of additives to control the size of the ice crystals, ultimately yielding functional materials with the desired pore size [71]. Isotropic ice‐templated materials are often applied in aerogels, fireproof materials, and thermal insulation materials, etc. [69, 72, 73].

#### 2.1.2 Unidirectional Ice‐Templating Strategy

For certain applications, microchannels are required for the transport of liquids or ions, giving rise to the development of unidirectional ice‐templating technology [74, 75]. This technique typically establishes a temperature gradient exclusively in the vertical direction, guiding ice crystals to grow continuously along this vertical axis and thereby forming through‐going microchannels [76, 77, 78, 79]. To generate such a temperature field, a common strategy is to immerse a high–thermal‐conductivity substrate (e.g., copper plate) directly into liquid nitrogen or place it on a thermoelectric Peltier cold stage [80, 81]. The combination of the ultralow temperature of liquid nitrogen and the high thermal diffusivity of the substrate rapidly cools the substrate to near the cold‐source temperature within minutes, while the air above the cold substrate remains at room temperature. This creates a steep, localized temperature gradient between the cold substrate and the surrounding air. Within this thermal field, the precursor solidifies via continuous ice‐crystal growth along the heat‐flow direction. After freeze‐drying and other structure‐preserving treatments, ice‐templated materials are obtained [82]. A typical feature of unidirectional ice‐templated materials is the presence of randomly distributed pores in the transverse plane and continuous channel structures along the longitudinal direction, as shown in Figure 2b [82, 83].

#### 2.1.3 Bidirectional Ice‐Templating Strategy

In nacre‐like structures, the lamellar microarchitecture is the key to achieving the combination of high strength and toughness [84, 85]. To replicate such lamellae of nacre, directional ice branches growth along a single longitudinal axis is insufficient, leading to the development of bidirectional ice‐templating technology [86, 87]. The fundamental principle of this technique is to establish temperature gradients along two directions in three‐dimensional space, enabling ice crystals to grow simultaneously in both directions to form ice platelets, while the non‐aqueous components are compressed and concentrated between them [88]. Subsequent freeze‐drying or other crosslinking methods yield bidirectionally ice‐templated materials [89, 90].

To create such a dual‐gradient thermal field, a wedge‐shaped substrate was proposed [91]. The base of the wedge is in contact with a cold source (liquid nitrogen, chilled alcohol, or a Peltier cooling plate). The substrate must have low thermal conductivity to generate a pronounced temperature gradient between the lower and upper edges of its inclined surface [92]. A precursor solution placed on the slope of the wedge thus experiences temperature gradients in both the tangential and normal directions of the inclined surface, promoting ice‐crystal growth along these two axes simultaneously. Typical samples prepared via bidirectional ice‐templating exhibit nacre‐like lamellar morphologies, as shown in Figure 2c [59]. The voids between the lamellae can be filled with resin or other materials to further enhance their mechanical performance.

#### 2.1.4 Radial Ice‐Templating Strategy

In the microarchitecture of biological tissues such as liver and bone, radial structures appear to be a result of biological selection and hold significant potential in biomedical applications [27]. Such radial architectures can also be achieved through ice‐templating. Radial ice‐templating technology requires the establishment of a temperature gradient along the circumferential–radial direction [93, 94, 95]. To achieve this, a cylindrical mold made of a high–thermal‐conductivity material (e.g., copper or aluminum material) can be used, with its bottom end immersed in liquid nitrogen. This setup creates a pronounced temperature difference between the cylinder wall and the central air, thereby establishing a clear radial temperature gradient [96]. During the freeze‐casting process, ice crystals grow inward from the circumference toward the center along the radial direction (Figure 2d) [97]. Subsequent freeze‐drying yields ice‐plated materials displaying a central, radially oriented structure.

#### 2.1.5 Ion‐Specific Ice Recrystallization Assisted Ice‐Templating Strategy

Ice recrystallization refers to the process during freezing in which initially formed ice crystals undergo rearrangement and coalescence, ultimately driving the extinction of minor crystals and the advancement of major crystal growth [98]. This process significantly influences the ice size in ice‐templating, thereby enabling control over the pore size of the resulting porous biomaterials. Since pore size is a critical factor determining the properties of porous materials, such as mechanical strength, permeability, and cell compatibility, controlling ice recrystallization offers a precise means of tailoring pore architecture.

**FIGURE 3:**
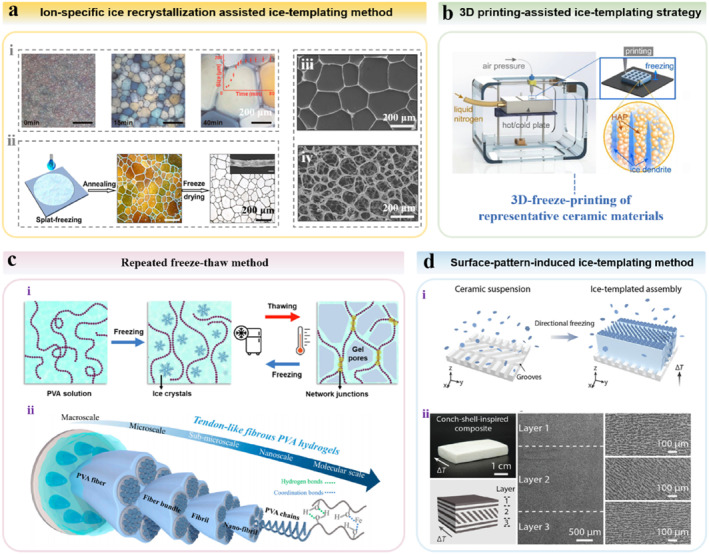
Physical ice‐templating strategy with external technical assistance. (a) Ice recrystallization assisted freeze‐casting strategy. (i) Ice recrystallization effect and (ii) freeze‐casting fabrication procedure [99]. (iii, iv) SEM showing the freeze‐casted structure [100]. (b) 3D printing‐assisted ice‐templating strategy [102]. (c) Repeated freeze‐thaw assisted ice‐templating strategy. (i) Fabrication procedures [108]. (ii) Typical hierarchical structure of PVA hydrogels produced by repeated freeze‐thawing [115]. (d) Surface‐pattern‐assisted ice‐templating strategy [118]. (i) Fabrication process. (ii) Optical and SEM images showing the structure. (ai, aii) Adapted with permission [99]. Copyright 2017, American Chemical Society. (aiii, aiv) Adapted under terms of the CC‐BY license [100]. Copyright 2017, The Authors, published by Springer Nature. (b) Adapted with permission [102]. Copyright 2019, Wiley‐VCH. (ci) Adapted with permission [108]. Copyright 2022, Elsevier. (cii) Adapted with permission [115]. Copyright 2025, The Authors, published by PNAS. (d) Adapted with permission [118]. Copyright 2022, Wiley‐VCH.

In the study by Wu et al. [99, 100] droplets were dropped from roughly 180 cm onto a precooled surface at −80°C for rapid freezing, and then subjected to annealing at −6°C for 45 min. This treatment produced polycrystalline ice layers containing grains of greater size when contrasted with unannealed counterparts, as shown in Figure 3a(i). Furthermore, altering both the ionic type and its concentration in the solution enabled the regulation of recrystallized ice dimensions from 27.4 ± 4.1 up to 277.5 ± 30.9 μm, with the ion‐regulating capability following the order I^−^ > Br^−^ > F^−^. Prolonging the annealing time also increased the ice crystal grain size. Using such size‐tunable recrystallized ice crystals as templates, they successfully fabricated porous cellular materials with controllable pore sizes, as shown in Figure 3a(ii–iv) [99, 100]. For instance, building upon the precise control over pore architecture enabled by ice‐recrystallization, where parameters like annealing and specific ions (e.g., I^−^) dictate final pore size, this strategy offers a powerful avenue for programming drug release kinetics in biomedical scaffolds. By tailoring the pore dimensions and connectivity, the diffusion rate of loaded therapeutics can be modulated, allowing designs that range from rapid initial release to sustained, long‐term delivery. This approach is particularly valuable for creating multifunctional tissue‐engineering scaffolds that combine structural support for tissue ingrowth with localized, controlled elution of bioactive molecules (e.g., growth factors or antibiotics), thereby enhancing regeneration while minimizing systemic side effects. Additionally, integrating this ice‐templating technique with stimuli‐responsive materials could further enable “smart” release systems triggered by specific microenvironmental cues. In future, ice‐recrystallization‐assisted freeze‐casting strategy is expected to be combined with precise thermal control, chemical regulation, and composite material technologies to expand applications in tissue engineering scaffolds, flexible electronics, filtration and separation, and energy materials, enabling high‐precision design and large‐scale production of materials with multiscale architectures [101].

#### 2.1.6 3D Printing‐Assisted Ice‐Templating Strategy

3D‐freeze‐printing is an advanced manufacturing technique that combines 3D printing with freeze‐casting [60]. 3D printing is an additive manufacturing process that builds three‐dimensional structures based on digital models by depositing materials layer by layer. It offers highly customizable shapes, high processing precision, and the ability to create structures of virtually unlimited complexity. Freeze‐casting, on the other hand, exploits the directional growth of ice crystals to program the material microstructure, enabling precise regulation of pore size, orientation, and interconnectivity.

The key advantage of 3D‐freeze‐printing lies in its dual‐level customization (Figure 3b) [102]: 3D printing provides flexible control over the macroscopic geometry and dimensions, while freeze‐casting fine‐tunes the microscopic pore architecture. This synergistic approach not only enables the rapid fabrication of complex porous structures but also allows the adjustment of thermal and electrical pathways, the design of anisotropic insulation channels, and improvements in both mechanical and electrical performance.

In related studies, this technology has been successfully applied to fabricate various material systems, including macroporous alumina scaffolds [103], MXene aerogels [104], graphene aerogels [105], and micro‐supercapacitors [106]. Incorporating carbon nanotubes (CNTs), graphene, or MXene into the printing slurry can significantly enhance the electrical conductivity and mechanical strength of the products, opening up promising applications in strain sensors [107], thermal management, catalysis, and impact absorption. In future, 3D‐freeze‐printing is expected to be integrated with multi‐material composites, precise thermal control, and functionally graded designs, driving its advancement toward higher performance and large‐scale production in fields such as electronic engineering, energy storage, and tissue engineering scaffolds.

#### 2.1.7 Repeated Freeze‐Thawing Assisted Ice‐Templating Strategy

The repeated freeze–thaw cycle is a strategy that partially replicates ice crystal structures through the formation and melting of ice crystals, thereby enabling physical crosslinking and structural regulation of materials [23]. Taking PVA freeze–thaw crosslinking as an example (Figure 3c) [108], an aqueous PVA solution is first frozen for a certain period. During ice crystal growth, polymer chains are expelled into the gaps between the ice crystals, generating compressive microstresses on the PVA molecular chains. This increases interchain contact, promotes chain entanglement and hydrogen bonding, and results in the formation of localized “entanglement nodes” and microcrystalline domains. Upon recovering to room temperature, the ice crystals melt, leaving behind a porous structure replicated from the ice crystal morphology. Multiple freeze–thaw cycles continuously generate new physically crosslinked regions, enhancing chain alignment and folding, and producing ice‐templated hydrogels with semicrystalline features. A variety of studies have shown that introducing other hydroxyl‐containing polymer chains, such as chitosan(CS) [109], hyaluronic acid (HA) [110], polyacrylic acid (PAA) [111], polyacrylamide (PAM) [112], and so on, into the PVA network can significantly strengthen hydrogen bonding within the system, accelerate the gelation process, and improve mechanical strength [103, 114, 115]. Based on these principles, a range of freeze–thaw treated materials have been fabricated, including PVA–xanthan gum hydrogels [116], PVA‐PAM hydrogels [112], and HA hydrogels [110], and calcium–PAA hydrogel [111]. In the future, the repeated freeze–thaw method is expected to be combined with nanomaterials, functional modifications, and gradient structural designs to develop ice‐templated hydrogels with high strength, multifunctionality, and precisely tunable microstructures, enabling broader applications in tissue engineering, flexible electronics, sensors, and drug delivery [2, 117].

#### 2.1.8 Surface‐Pattern‐Assisted Ice‐Templating Strategy

The surface‐pattern‐assisted ice‐templating strategy is a novel method for precisely directing ice crystal growth by constructing complex temperature fields. The core concept involves using a high‐thermal‐conductivity material (with a thermal conductivity at least one order of magnitude higher than that of air, 0.026 W m^−1^ K^−1^) to fabricate a patterned substrate, onto which pits are engraved via laser etching to create intricate surface patterns. These pits generate localized temperature gradients between the high‐thermal‐conductivity patterned regions and the low‐thermal‐conductivity air pockets, thereby guiding ice crystals to grow in specific directions and enabling the formation of complex oriented structures.

This strategy features high controllability and good design flexibility, allowing for multi‐scale orientation control from the macroscopic to the microscopic level within the material, thus imparting excellent mechanical and functional properties to the final materials. For example, Li et al. [118] designed multiple groove regions to fabricate ice‐templated materials with a layered, nacre‐like hierarchical structure, exhibiting multi‐level microstructures along with outstanding toughness and impact resistance (Figure 3d). This method shows great promise in various fields, such as high‐performance structural materials, bioinspired composites, protective armor, and energy‐absorbing materials, etc. In the future, surface‐pattern‐assisted ice‐templating is expected to be integrated with computational design, variable temperature field control, and multi‐material composite strategies to achieve even more complex and precise control over ice crystal growth, driving innovation in ice‐templated materials for aerospace, and biomedical applications [119].

### 2.2 Chemical Ice‐Templating Strategy

#### 2.2.1 Ice‐Confined In Situ Cryopolymerization Strategy

**FIGURE 4:**
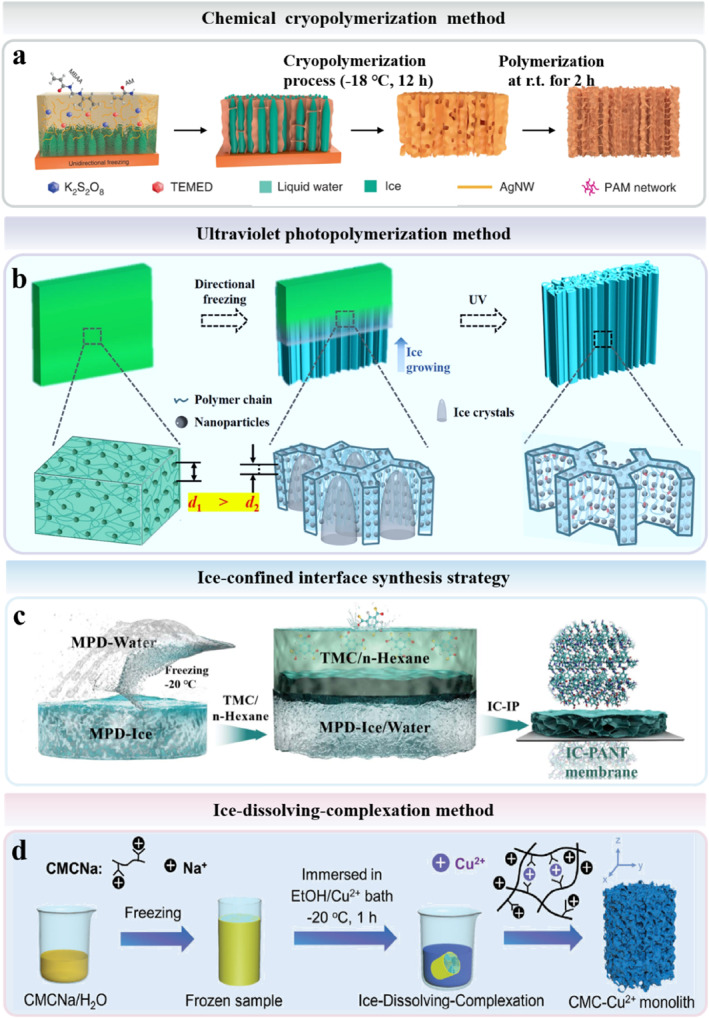
Chemical ice‐templating strategy. (a) Ice‐confined in situ cryopolymerization strategy [120]. (b) Ice‐confined photopolymerizaton strategy [125]. (c) Ice‐confined interface synthesis strategy [129]. (d) Ice‐dissolving‐complexation strategy [139]. (a) Adapted under terms of the CC‐BY license [120]. Copyright 2022, The Authors, published by Springer Nature. (b) Adapted under terms of the CC‐BY license [125]. Copyright 2022, The Authors, published by Springer Nature. (c) Adapted with permission [129]. Copyright 2023, The Authors, published by AAAS. (d) Adapted with permission [139]. Copyright 2021, Wiley‐VCH.

The ice‐confined in situ cryopolymerization strategy is a novel method that incorporates chemical reactions into ice‐templating technology to directly lock ice crystal structures under low‐temperature conditions. The process generally involves two stages (Figure 4a) [120]. First, polymerizable monomers (e.g., acrylamide) and crosslinkers (e.g., *N*,*N*′‐methylenebisacrylamide) were dissolved in water and frozen at low temperatures. During ice crystal growth, the monomers and crosslinkers are expelled into the concentrated liquid phase between the ice crystals, where chemical crosslinking reactions occur within this confined space, forming an initial three‐dimensional network structure. Subsequently, the sample was returned to room temperature for in situ polymerization, further solidifying and stabilizing the network.

The underlying principle is to utilize the confinement effect of ice crystal growth, forcing polymerization to occur in the intercrystalline regions. The ice phase acts as a template to define the pore architecture of the material, while low‐temperature in situ chemical crosslinking locks this structure in place before the ice melts, achieving a synergistic effect between physical templating and chemical fixation [121]. Compared with traditional purely physical approaches such as freeze‐drying or repeated freeze–thaw cycles, this strategy significantly shortens processing time, improves efficiency, and reduces the risk of structural collapse upon template melting, while imparting excellent mechanical and other functional properties to the material.

In the study by Wang et al. [120] this method was successfully employed to fabricate ice‐templated hydrogels with interconnected cellular network structures, exhibiting outstanding mechanical strength and electrical conductivity, thereby validating its feasibility and advantages in structural stabilization and performance enhancement. In the future, this strategy holds great potential for expansion to a wider range of monomer systems and for integration with conductive polymers, nanofillers, and functional modification techniques to develop high‐strength, multifunctional, and structurally tunable ice‐templated materials for applications in flexible electronics, sensors, energy storage, biomedical materials, etc.

#### 2.2.2 Ice‐Confined Photopolymerizaton Strategy

The ice‐confined photopolymerization strategy is based on combining ultraviolet (UV) photopolymerization with freeze‐casting technology. In this approach, under low‐temperature frozen conditions, UV light is used to initiate free‐radical polymerization, solidifying polymerizable components that are segregated and concentrated in the interstices between ice crystals into a network structure, thereby locking the ice crystal morphology before it melts [62]. The specific procedure involves first preparing a solution of monomers, crosslinkers, and a photoinitiator, followed by freezing at low temperature so that ice crystals form within the system and expel the polymerizable components into the intercrystalline regions. The frozen sample was then exposed to UV light to trigger polymerization, forming a solidified network structure within the ice crystal gaps. Finally, when the temperature is returned to room temperature, the ice melts and is removed, leaving a solidified ice‐templated structure [122, 123].

The fundamental principle of this strategy is to exploit the confinement effect generated by ice crystal growth, which restricts the polymerization space to the intercrystalline regions, and to achieve rapid structural fixation through UV‐initiated polymerization. This method offers the advantages of simple operation, high fabrication efficiency, and suitability for wet‐state final products, while avoiding structural collapse that can occur during drying or melting. In research examples, Bai et al. [124] employed UV‐initiated ice‐confined photopolymerizaton strategy to fabricate a temperature‐responsive composite hydrogel possessing a two‐level lamellar morphology extending across the micro‐ and nanoscale ranges, closely resembling the structure of natural nacre, and exhibiting excellent mechanical properties and temperature responsiveness. In another example, Miao et al. [125] designed an ice‐templated structural colored membrane by photopolymerization of a PEGDA suspension containing SiO_2_ nanoparticles under frozen conditions, producing a multiscale hierarchical porous colored hydrogel with thermoresponsive color‐changing capability (Figure 4b).

In the foreseeable future, the ice‐confined photopolymerization strategy is expected to be integrated with functional monomers, nanofillers, stimuli‐responsive components, and controllable temperature‐field technologies to develop multifunctional, high‐performance ice‐templated materials with precisely tunable morphologies, expanding their potential applications in flexible electronics, optical devices, sensors, and tissue engineering.

#### 2.2.3 Ice‐Confined Interface Synthesis Strategy

The ice‐confined interface synthesis strategy is an attractive method for fabricating functional membrane materials [126]. Its core concept is to utilize the confinement and gradual release effects of ice crystals to achieve precise control over reactions during interfacial polymerization [127, 128]. The process involves first freezing an aqueous solution containing one reactant (e.g., an *m*‐Phenylenediamine (MPD) solution) at subzero temperatures to form a frozen phase. Then, at room temperature, a pre‐chilled solution of the other reactant in an organic phase (e.g., a TMC solution dissolved in n‐hexane) was gently applied onto the surface of the MPD ice. As the ice progressively melts, the aqueous reactant is slowly released to the reaction interface, where it reacts with the organic‐phase reactant via interfacial polymerization (Figure 4c) [129].

The fundamental principle lies in the fact that the ice crystals not only provide an initial low‐temperature reaction space but also enable precise regulation of reactant diffusion rates and reaction kinetics through the gradual melting process. This allows for fine‐tuning of membrane structure and performance during formation [130]. Compared with conventional interfacial polymerization, this strategy introduces more controllable microstructures into the membrane, improving molecular channel distribution and alignment.

The advantages of the ice‐confined interface synthesis strategy include highly controllable reaction processes, the ability to precisely regulate reactant release rates, and the enhancement of pore structure and molecular sieving performance. In related researches, Zhang et al. [129] successfully applied this method to fabricate ion‐selective polyamide membranes that maintained excellent selectivity while achieving significantly higher water permeability, effectively distinguishing smaller ions such as chloride from larger ones like sulfate. In the near future, the ice‐confined interface synthesis strategy is expected to be extended to the preparation of gas separation membranes, ion‐selective membranes, and biomolecular separation membranes. Furthermore, by integrating with nanomaterial modification and bioinspired structural design, it has the potential to produce advanced membrane materials with high performance and tunable functionalities, enabling broader applications in seawater desalination [131, 132, 133, 134], energy conversion [135], and environmental purification [136, 137].

#### 2.2.4 Ice‐Dissolving‐Complexation Strategy

The ice‐dissolving‐complexation strategy is a useful method for fixing ice‐templated structures without the need for freeze‐drying, designed to address the low efficiency caused by the prolonged freeze‐drying process in traditional freeze‐casting [138]. The procedure involves first completely freezing a polyelectrolyte solution (e.g., sodium carboxymethyl cellulose solution) to form a frozen bulk. The frozen material is then directly immersed into a pre‐cooled metal ion/organic solution (e.g., Cu^2+^/ethanol solution). During this process, the ice crystals rapidly dissolve in the organic solvent, while the polyelectrolyte (sodium carboxymethyl cellulose) simultaneously undergoes complexation with the metal ions (Cu^2+^). This reaction promptly locks in the pores or aligned channels created by the ice crystals, resulting in a stable monolithic polyelectrolyte material (Figure 4d) [139].

The fundamental principle lies in the simultaneous action of ice dissolution in a low‐temperature organic solvent (such as ethanol) and the complexation between metal ions and the polyelectrolyte. At the moment the ice crystals disappear, the complexation effect supports and stabilizes the original microstructure [140, 141, 142]. The advantages of this method include the ability to remove the ice template and immobilize the structure in a single step, a significantly shorter ice‐removal time, which is 30–50 times faster than conventional freeze‐drying, and the prevention of structural collapse that can occur during vacuum drying. Looking ahead, the ice‐dissolving‐complexation strategy has the potential to be extended to a wider range of polyelectrolyte systems and functional metal ions, enabling the efficient fabrication of multifunctional porous materials with precisely controlled structures.

## 3 Progress of Ice‐Templating Technology in Tissue Engineering Applications

In cell culture and regenerative medicine, scaffold systems provide a dynamic structural environment that supports cell attachment, proliferation, differentiation, and tissue formation over time [36, 143, 144, 145]. Precisely engineered oriented micro‐scale pores within these scaffolds greatly enhance nutrient diffusion over long distances and enable cell growth in a directional manner. The freeze‐casting technique, or ice‐templating, has been broadly employed to construct porous scaffolds with tunable architectures, specifically tailored to meet the complex demands of three‐dimensional biological culture systems and tissue engineering [85, 146]. The unique advantage of ice‐templating in tissue engineering lies in its ability to controllably fabricate three‐dimensional structures that integrate macroporosity, structural alignment, and mechanical strength. These characteristics directly address the critical requirements for tissue regeneration, including cell infiltration, vascularization, mechanical support, and structural guidance. For instance, many native tissues (e.g., bone, cartilage, nerve, and muscle) possess distinct anisotropic structures, such as the Haversian canals in osteons and the bundled architecture in nerves. Through directional freezing, ice‐templating can precisely create layer‐ or tube‐like pore channels that are highly aligned, long‐range ordered, and with tunable pore sizes (typically 1–200 μm). This unique architecture physically guides directional cell migration, alignment, and differentiation, which is a feat difficult to achieve with many randomly porous scaffolds or nanofiber mats.

Furthermore, a core challenge in tissue engineering is to construct three‐dimensional, large‐volume scaffolds that enable uniform cell distribution and vascular network integration throughout their interior. The macropores (typically > 50 μm) and excellent pore interconnectivity formed via ice‐templating provide the necessary physical space for cell migration, proliferation, and subsequent vascular ingrowth into the scaffold's depth, effectively preventing the issue of cells being confined only to the surface due to insufficient pore size.

In addition, ice‐templated structures offer outstanding mechanical properties and structural stability. The pore walls of ice‐templated scaffolds are typically dense, and the aligned structure formed along the freezing direction results in mechanically reinforced architectures. This leads to superior compressive and tensile performance compared to many flexible fibrous scaffolds, which is crucial for repairing load‐bearing tissues (e.g., bone and cartilage) by providing temporary mechanical support that matches the host tissue's mechanical environment.

Moreover, ice‐templating can serve as a fundamental fabrication module and be integrated with other technologies such as 3D printing and microfluidics. For example, combining the macroscopic shape defined by 3D printing with the micro‐scale anisotropic channels created by ice‐templating enables cross‐scale structural biomimicry, offering a novel strategy for constructing complex tissues and organs.

**FIGURE 5:**
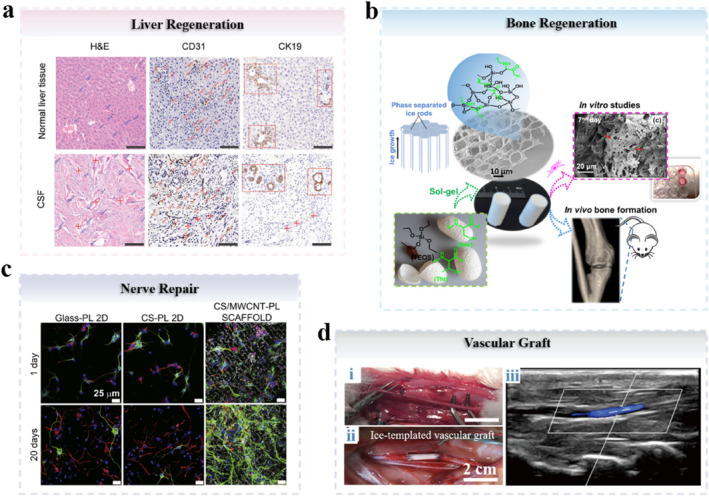
Examples of ice‐templated scaffolds for various tissue engineering applications. (a) Ice‐templated chitosan‐fibers cryogel (CSF) for in situ liver regeneration [147]. (b) Ice‐templated silica‐silk fibroin bioaerogel for in vivo bone formation [148]. (c) Ice‐templated chondroitin sulphate/MWCNT scaffold for embryonic neural progenitor cell differentiation [149]. (d) Ice‐templated vascular graft for revasculature [150]. Optical images of (i) Native vessel and (ii) ice‐templated vascular grafts in rabbits. (iii) In situ sonographic images of ice‐templated vascular grafts. (a) Adapted with permission [147]. Copyright 2023, Wiley‐VCH. (b) Adapted with permission [148]. Copyright 2019, American Chemical Society. (c) Adapted with permission [149]. Copyright 2014, Elsevier. (d) Adapted with permission [150]. Copyright 2019, American Chemical Society.

Compared to other scaffold fabrication techniques (e.g., electrospinning), ice‐templating excels at creating scaffolds with highly aligned, macroporous structures, which are crucial for guiding deep cell infiltration and tissue vascularization in applications like bone repair. Its key advantages include superior mechanical strength and the ability to direct cell growth along controlled channels. However, it offers less nanoscale topographic control for cell adhesion compared to the fibrous networks produced by electrospinning (refer to Table 2 for detailed comparison). In recent years, based on the above advantages, a variety of scaffolds have been proposed, such as freeze‐casted chitosan‐fibers hydrogel (CSF) for in situ liver regeneration [147], ice‐templated silica‐silk fibroin bioaerogel for in vivo bone formation [148], ice‐templated chondroitin sulphate/MWCNT scaffold for embryonic neural progenitor cells differentiation [149], and ice‐templated vascular graft for revasculature (Figure 5) [150].

**TABLE 2:** Comparison between ice‐templating and electrospinning technologies.

	Ice‐templating	Electrospinning
Core principle	Directional freezing of a solution/suspension, where growing ice crystals exclude solutes, forming a porous structure after sublimation.	A polymer solution or melt is stretched and solidified by a high‐voltage electrostatic field, collected as nanofibers.
Typical structure	Macroporous/Lamellar pores (pore size typically 1–200 μm), highly aligned channels, dense pore walls.	Nanofibrous network (fiber diameter 50–1000 nm), high specific surface area, high porosity but relatively smaller pores (typically < 10 μm).
Key advantages	Highly tunable pore size and channel architecture (via freezing rate, direction, etc.). Excellent mechanical properties, especially along the pore alignment. Broad material compatibility (ceramics, polymers, composites, etc.). Favorable for deep cell infiltration and tissue vascularization (due to macroporosity).	1. Extremely high specific surface area, beneficial for cell adhesion and mass transfer. 2. Can mimic the nanofibrous topography of the native extracellular matrix (ECM). 3. Easily tunable fiber composition and morphology (e.g., core‐shell). 4. Relatively simple setup and ease of processing.
Limitations	1. Limited resolution, making it difficult to create nano‐scale features. 2. Time‐consuming process (long freezing and sublimation times). 3. Sensitive to solution properties (e.g., concentration, viscosity). 4. Often requires post‐processing (e.g., crosslinking, sintering).	1. Relatively small pore size may restrict 3D cell infiltration and migration. 2. Poor pore interconnectivity when fibers are densely packed. 3. Generally weaker mechanical strength (especially for non‐woven mats). 4. Frequent use of organic solvents raises potential toxicity concerns.
Typical applications	Bone tissue engineering (load‐bearing, large pores), nerve guidance conduits (aligned channels), filtration/catalytic carriers (directional transport).	Soft tissue engineering (skin, vascular), drug delivery carriers, wound dressings, sensors.

The microstructure of ice‐templated materials for tissue engineering applications can be smartly regulated during the fabrication process. For instance, by designing substrate materials with differential thermal conductivity and specific freezing strategy, the size and orientation of ice crystals and structural parameters of ice‐templated materials can be precisely tailored. This enables the creation of tissue engineering scaffolds with specific structures and biological functions (Table 3).

**TABLE 3:** Quantitative relationships between structural parameters of ice‐templated biomaterials and their biological performance.

Structural parameters	Performance link	Quantitative influence and rationale for biological performance
Pore size and distribution	Cell infiltration and tissue ingrowth	∼20–150 μm: Optimal for dermal fibroblast invasion (wound healing). > 200 μm: Favors osteoblast migration & bone formation (bone regeneration). Narrow distribution ensures uniform cellular response.
Pore interconnectivity	Mass transport and vascularization	Interconnectivity > 90% is critical for efficient nutrient/waste diffusion. Direct correlation with in vitro capillary network length and in vivo blood vessel density.
Porosity	Mechanical support versus bioactivity	Porosity ∼85%–95%: Maximizes surface area for cell attachment but reduces compressive modulus (e.g., ∼1–10 kPa for soft tissue mimics). Porosity ∼70%‐85%: Enhances modulus (∼10–100 MPa) for pre‐calcified bone scaffolds.
Pore wall topography (nano/micro‐features)	Cell adhesion and differentiation	Nanofiber decoration on pore walls increases surface area by 3–5x, enhancing protein adsorption. Correlates with increased cell adhesion density (cells/mm^2^) and upregulated osteogenic/neuronal marker expression.
Structural anisotropy (aligned channels)	Contact guidance and directional tissue growth	Anisotropic scaffolds with channel alignment > 80% guide axonal growth in nerve repair (increased neurite length vs. isotropic scaffolds). In tendon/ligament engineering, anisotropy increases cell alignment index and collagen fiber orientation.

### 3.1 Scaffold for 3D Cell Culture

In recent years, freeze‐casting has emerged as a powerful strategy for constructing 3D cell culture scaffolds [94, 151]. Compared to conventional scaffold fabrication techniques, the ice‐templating method offers several remarkable advantages for 3D cell culture. Firstly, the controllable ice crystal growth enables precise tuning of pore size and orientation based on specific design requirements. Secondly, this process operates under low‐temperature conditions and avoids the use of heat or toxic reagents, thereby preventing denaturation of thermally sensitive biomolecules such as collagen and eliminating the incorporation of cytotoxic chemicals [152]. As a result, it is considered a green, safe, and biocompatible treatment approach. Additionally, the ice crystal‐induced, directionally oriented channels enhance nutrient and oxygen transport throughout the scaffold, supporting a microenvironment conducive to cell proliferation [153, 154].

**FIGURE 6:**
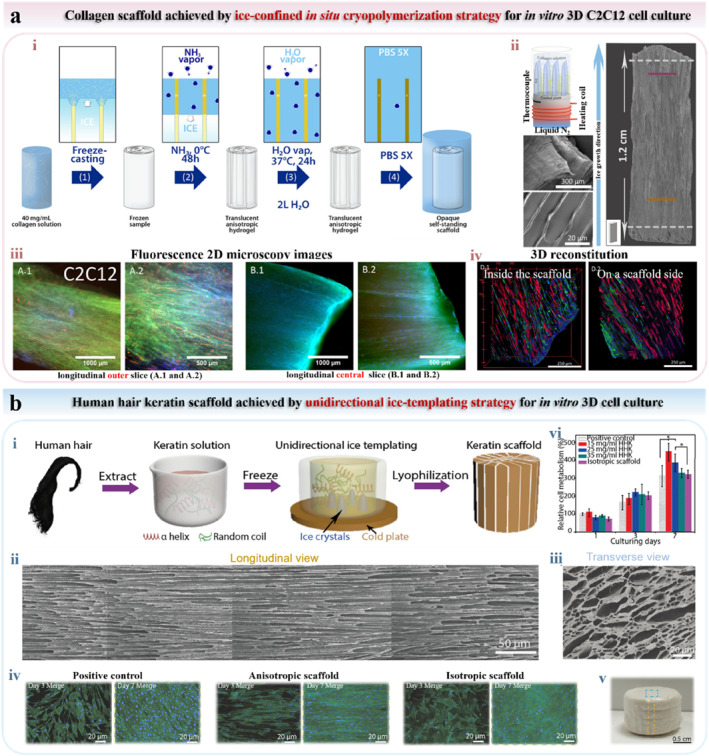
Ice‐templated scaffold for 3D cell culture. (a) Ice‐templated collagen scaffold for cell culture [162]. (i) Preparation and (ii) morphology. (iii) Fluorescence 2D images of C2C12 proliferation (iv) 3D reconstitution. (b) Ice‐templated human hair keratin scaffold for in vitro 3D cell culture [163]. (i) Preparation process. (ii) Longitudinal structures and (iii) transverse structure of the ice‐templated keratin scaffold. (iv) Cytocompatibility comparison of the conventional tissue culture polystyrene (positive control), isotropic ice‐templated scaffold and directional ice‐templated anisotropic scaffold. (v) Optical image of the ice‐templated keratin scaffold. (vi) Comparison of relative cell metabolism among the positive control, isotropic ice‐templated scaffolds, and directional ice‐templated anisotropic scaffolds (tested at different concentrations). (a) Adapted with permission [162]. Copyright 2019, American Chemical Society. (b) Adapted with permission [163]. Copyright 2021, Wiley‐VCH.

Since 3D cell culture systems aim to replicate the physiological environment in vivo, higher demands are placed on the structural and compositional features of scaffolds [155, 156, 157]. Therefore, materials used in such systems must be biocompatible, and the pore size should be appropriately matched to the dimensions of the target tissue or organ. Suitable materials include hydroxyapatite, gelatin, collagen, chitosan, and silk fibroin [96, 158], which are hydrophilic macromolecules known for their low immunogenicity. By carefully adjusting parameters such as freezing temperature, freezing front velocity, precursor concentration, and additives, the pore size and spatial architecture of the scaffold can be precisely controlled. With such fine‐tuned structural customization, the resulting scaffolds can be tailored to mimic the desired tissue microenvironments, offering broad potential in various tissue engineering applications [159, 160, 161]. Rieu et al. [162] modified the topological morphology of freeze‐casted collagen scaffolds using ammonia vapor (Figure 6a). Upon contact with ice, the ammonia gradually lowers the freezing point and facilitates early‐stage collagen fibril formation, all while retaining the microscale architecture established by ice‐templating. Fibrous collagen matrix scaffolds generated by this process exhibit outstanding mechanical performance and feature a hierarchical microstructure: layered pores aligned along the freezing trajectory combined with regularly spaced ridges along the pore walls. The 3D in vitro cultivation of C2C12 myoblasts showed that cells migrated directionally along lamellar pores within the scaffold, implying strengthened cell–matrix interactions.

Human hair keratin (HHK), known for its outstanding biocompatibility and bioactivity, has been successfully employed as a template material for soft tissue regeneration, with significant potential in tissue engineering. Although HHK scaffolds have demonstrated promising biological performance in tissue regeneration, most of them lack finely tuned microstructural design, limiting their ability to replicate the anisotropic properties of soft tissues such as muscles, tendons, and ligaments. Fortunately, directional freeze‐casting offers a powerful solution to this limitation. Zhao et al. [163] extracted keratin from human hair and employed directional freeze‐casting to fabricate a 3D scaffold with well‐defined microarchitecture (Figure 6b). These scaffolds achieved highly aligned channels, significantly enhancing the mechanical strength along the channel orientation. Cell migration assays revealed that, compared with the non‐templated control, the ice‐templated 3D aerogel exhibited a remarkable enhancement in cellular migratory distance, which is up to a 250% increase. The HHK scaffolds demonstrated excellent biocompatibility and bioactivity by supporting cell adhesion, spreading, and proliferation in vitro. These findings indicate that keratin materials could be employed in tissue engineering for the repair of anisotropic soft tissues, including tendons and ligaments.

### 3.2 Wound Healing

**FIGURE 7:**
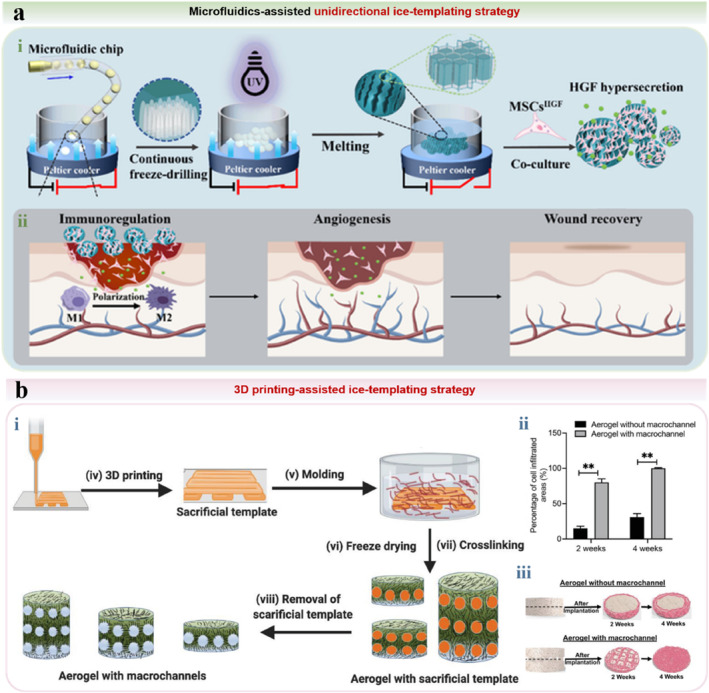
Wound healing. (a) Freeze‐casted microcarriers from microfluidics‐assisted unidirectional ice‐templating strategy for wound healing [39]. (i) Fabrication process. (ii) Wound healing process. (b) Freeze‐casted aerogel scaffolds from 3D printing‐assisted ice‐templating strategy for cellular infiltration [168]. (i) Fabrication procedure. (ii) Percentages of cell‐infiltrated areas. (iii) Comparison of cell distributions to show improved cellular infiltration in the freeze‐casted aerogel scaffolds with 3D printed microchannel. (a) Adapted under terms of the CC‐BY license [39]. Copyright 2025, The Authors, published by AAAS. (b) Adapted with permission [168]. Copyright 2021, Wiley‐VCH.

Scaffolds for wound healing are typically loaded with growth factors, stem cells, antibacterial or anti‐inflammatory agents, and extracellular matrix (ECM)‐like components to accelerate tissue repair, prevent infection, and restore the structural integrity of damaged tissues [164, 165, 166]. Notably, directionally aligned microchannels within the scaffold provide pathways for nutrient and waste transport, enhance cell migration, and foster angiogenic responses. Aerogel scaffolds fabricated via freeze‐casting have demonstrated great potential as wound healing and tissue regeneration platforms due to their high surface area, porosity, and biomimetic architectures. For example, Cai et al. [39] proposed a directional freeze‐casted microsphere system by combining microfluidics and directional freeze‐casting technologies to encapsulate mesenchymal stem cells (MSCs) overexpressing hepatocyte growth factor (HGF) for enhanced wound healing (Figure 7a). Uniform microdroplets were first generated via a microfluidic technique. Then, microdroplets were subjected to gradient freeze‐casting and in situ photopolymerization to form the desired microspheres with aligned micron‐scale channel structures. Owing to their biocompatible chemical composition and oriented architecture, these freeze‐casted microspheres enabled efficient encapsulation and sustained release of MSCs, maintained prolonged HGF secretion, promoted cell migration, and supported neovascularization, ultimately improving the efficiency of skin wound repair to 97.21% ± 2.39% by day 11.

Due to its structural controllability and tunable pore size, freeze‐casting has recently emerged as an ideal tool for constructing micro‐structured scaffolds required in wound healing applications. However, adjusting the porosity of scaffolds by varying the freezing temperature within a limited range often yields unsatisfactory outcomes in tissue regeneration, primarily due to suboptimal pore sizes and architectures [28]. Non‐ideal microstructures can lead to poor cellular infiltration and uneven cell distribution within the scaffolds, thereby impeding cell migration and tissue regeneration [167]. To address this, scaffolds with optimized channel architectures are essential for aiding cell penetration and sustaining uniform nutrient and oxygen availability. To this end, John et al. [168] developed a hybrid strategy that combines 3D‐printed sacrificial templates with ice‐templating to construct scaffolds containing both macro‐ and microchannels (Figure 7b). By inversely replicating the 3D‐printed template, single or multilayered macrochannels can be created, while the directionally freeze‐casted microchannels serve as interconnecting pores between these large channels. The resulting macro‐/microchannel structure within the aerogel scaffold significantly improved the infiltration of osteoblasts in vitro. Furthermore, freeze‐cast scaffolds functionalized with the VEGF‐mimicking QK peptide supported endothelial cell‐driven formation of microvascular networks, indicating their applicability in tissue healing and regenerative medicine.

### 3.3 Bone Regeneration

Cell therapy is an advanced regenerative medicine strategy for bone repair [169, 170], aiming to promote the healing and regeneration of bone defects by transplanting cells with osteogenic potential. Commonly used cell types include bone marrow mesenchymal stem cells (BMSCs), induced pluripotent stem cells (iPSCs), and so on. These cells can differentiate into osteoblasts and secrete various growth factors, thereby not only promoting new bone formation but also supporting angiogenesis to improve the local microenvironment. In both clinical and experimental studies, cell therapy is often combined with biomaterial scaffolds, such as hydroxyapatite or collagen‐based scaffolds, to provide 3D structural support and enhance cell adhesion and survival. Compared with traditional bone grafting, cell therapy can reduce donor site morbidity and is suitable for large bone defects or poorly healing fractures. Despite its great promise, challenges remain in bone repair applications, with future research focusing on optimizing cell preparation, precise delivery, and synergistic integration with biomaterials and bioactive factors to achieve more efficient and controllable bone regeneration.

**FIGURE 8:**
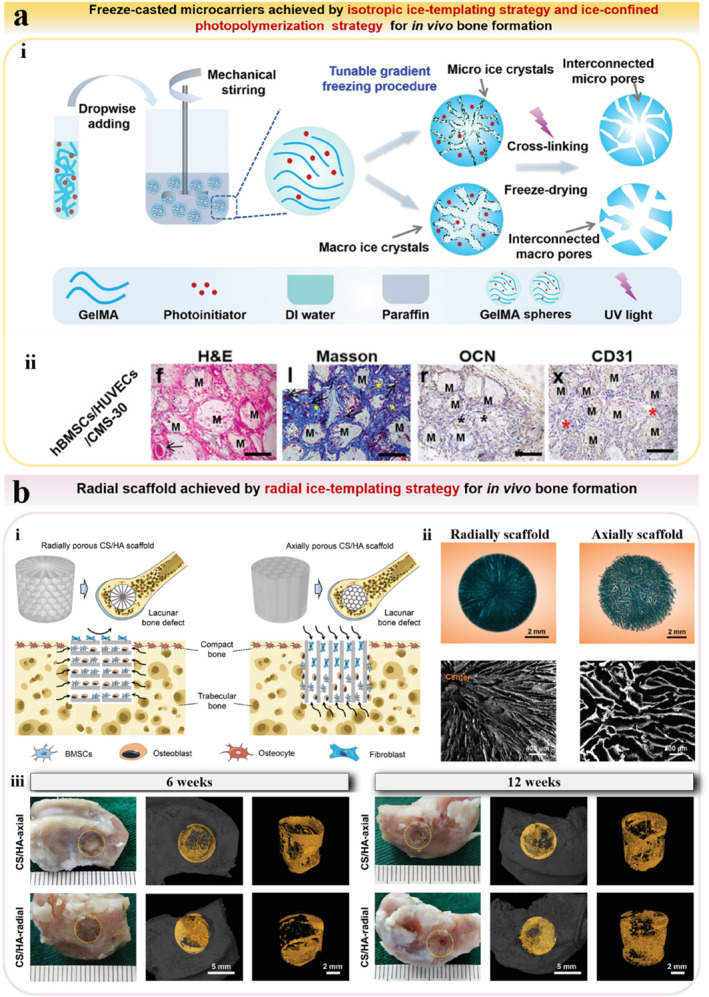
Bone regeneration. (a) Freeze‐casted microcarriers for in vivo bone formation [171]. (i) Illustration of fabrication for freeze‐casted microcarriers. (ii) Immunohistochemistry staining for evaluating bone formation. (b) Radial ice‐templated scaffold for in vivo bone formation [174]. (i) Schematic illustration of bone formation, (ii) morphology, (iii) in vivo experiments. (a) Adapted with permission [171]. Copyright 2021, Wiley‐VCH. (b) Adapted with permission [174]. Copyright 2022, Wiley‐VCH.

Among these approaches, ice‐templated hydrogels have attracted considerable attention for cell delivery due to their highly interconnected porous networks and strong mechanical stability. For example, to address the low efficiency of cell delivery in cell therapy, Yuan et al. [171] developed porous shape‐memory ice‐templated hydrogel microspheres based on gelatin methacrylate (GelMA), as shown in Figure 8a. By combining emulsification with a gradient cooling cryogelation method, the pore size of the ice‐templated microspheres can be precisely tuned by adjusting the cooling time. Notably, 30‐min gradient cooling produced microspheres with an optimal pore size of 15.5 ± 6.0 μm. This structure facilitates cell adhesion and sustained proliferation while protecting cell viability during injection. In vivo, a 1:1 mixture of ice‐templated hydrogel microspheres loaded with the two cell types was subcutaneously injected into nude mice, resulting in vascularized bone‐like tissue with high expression of osteocalcin (OCN) and cluster of differentiation 31 (CD31). These findings indicate that ice‐templated GelMA microspheres, with their tunable pore size, excellent biocompatibility, and multi‐cell delivery capability, offer an ideal injectable carrier for functional tissue regeneration.

In the process of bone repair, the microstructure of the scaffold plays a critical role in determining the repair outcome [172]. An ideal scaffold should possess appropriate pore size and interconnectivity to facilitate cell infiltration and migration, ensure efficient exchange of nutrients and metabolic waste, and provide a favorable environment for extracellular matrix deposition. Moreover, scaffolds with specific morphologies can effectively prevent the invasion of surrounding fibroblasts and other non‐osseous tissues, thereby avoiding interference with the bone regeneration process [173]. However, fabricating scaffolds that meet these multiple structural requirements remains technically challenging. On one hand, conventional fabrication methods have limitations in controlling pore size distribution, orientation, and hierarchical architecture; on the other hand, the microstructural demands vary depending on anatomical location and defect type, necessitating highly customized scaffold designs.

Freeze‐casting technology, with its excellent controllability over pore architecture, offers a powerful solution to this challenge. By regulating the growth direction, rate, and morphology of ice crystals, it is possible to precisely design the pore size, interconnectivity, and orientation of the scaffold at both macro‐ and micro‐scales. This method not only enables the creation of hierarchical pore structures resembling those of natural bone but also allows customization of scaffold microstructures to meet specific clinical requirements, thereby optimizing cellular behavior and promoting bone regeneration. For example, Jiang et al. [174] developed and fabricated an ice‐templated bone repair scaffold with a radially oriented porous structure, aiming to simultaneously promote osteoblast adhesion, proliferation, and migration while blocking the infiltration of non‐osteogenic cells and fibrous tissue, as shown in Figure 8b. Constructed via radial freeze‐casting, the scaffold's pore size and orientation were precisely controlled by adjusting the temperature gradient. Chitosan, with excellent biocompatibility, was selected as the organic matrix, while hydroxyapatite was incorporated as the inorganic reinforcement. In vitro and in vivo evaluations confirmed the scaffold's excellent compatibility with biological systems, guided cells deep into the scaffold along radial pores, and effectively prevented surrounding tissue invasion. In a cavitary bone defect animal model, its osteogenic performance was significantly superior to that of axially porous scaffolds, highlighting its unique potential for repairing plate‐like and cavitary bone defects. During fracture treatment or defect reconstruction, surgeons often require patient‐specific scaffolds with well‐defined, directionally aligned channels to facilitate osteogenesis and vascular ingrowth. The ice‐templating approach offers a versatile route to generate such anisotropic architectures that conform to the geometry of the defect site. Thus, freeze‐casting provides unique advantages and broad application prospects for the fabrication of high‐performance, patient‐specific bone repair scaffolds.

### 3.4 Nerve Conduits

**FIGURE 9:**
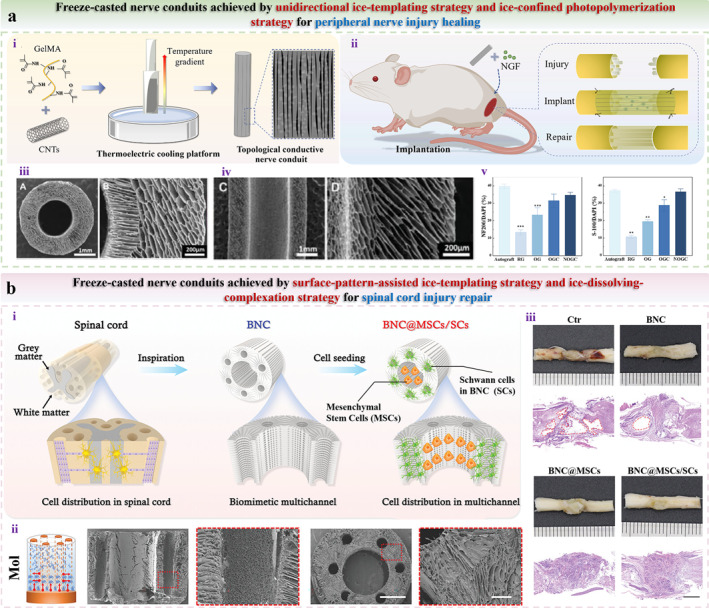
Freeze‐casted nerve conduits. (a) Freeze‐casted nerve conduits for peripheral nerve injury healing. (i, ii) Illustration of (i) fabrication and (ii) of healing performance [182]. (iii, iv) Typical SEM images of freeze‐casted nerve conduits [183]. (v) Quantitative analysis of animal experiments [182]. (b) Freeze‐casted nerve conduits for spinal cord injury repair [186]. (i) Fabrication procedure. (ii) Typical SEM images. (iii) Repairing performance. (ai, aii, av) Adapted under terms of the CC‐BY license [182]. Copyright 2025, The Authors, published by Wiley‐VCH. (aiii, aiv) Adapted with permission [183]. Copyright 2018, Springer Nature. (b) Adapted with permission [186]. Copyright 2024, Wiley‐VCH.

Peripheral nerve injury (PNI) is a globally prevalent condition that imposes a significant medical and economic burden on society [175]. Severe PNI often results in the disruption of communication between the central nervous system and peripheral tissues, thereby impairing signal transmission during motor functions [176]. In recent years, various strategies have been proposed for PNI treatment, among which the use of bioactive molecules such as nerve growth factors (NGF) and other neurotrophins has shown positive effects on nerve regeneration [177]. Despite these advances, pharmacological treatment alone is typically insufficient for repairing long‐gap nerve defects. In contrast, nerve guidance conduits (NGCs) derived from diverse biomaterials have emerged as an effective approach for promoting regeneration across nerve gaps [178, 179]. Notably, NGCs combined with therapeutic agents have been employed to enhance neuronal differentiation and migration at the injury site, thereby improving regenerative outcomes [180, 181]. However, many existing NGCs lack precise micro/nano‐structural designs to guide axonal extension, and they often neglect the provision of an optimal electrical microenvironment required for neuronal growth, leading to suboptimal regeneration and functional recovery. To address these limitations, Zhang et al. [182] proposed ice‐templated conductive nerve conduits fabricated via directional freeze‐casting technology for peripheral nerve regeneration (Figure 9a). Typical SEM images of the ice‐templated conduits are shown in Figure 9a(iv) [183]. The unique aligned topological structure of the conduits facilitates directional axonal growth within confined spaces. Both in vitro and in vivo experiments demonstrated that the fabricated ice‐templated conductive nerve conduits offer the following advantages: topological guidance of axonal outgrowth, electrical conductivity to stimulate cell proliferation and differentiation, and NGF release to promote neuronal differentiation (Figure 9a(v)). These findings highlight the clinical potential of such nerve conduits in enhancing peripheral nerve regeneration.

In addition to its application in peripheral nervous injury healing, freeze‐casting technology has also shown great promise in the repair of spinal cord central nervous system injuries. The functionality of spinal cord tissue heavily relies on the integrity of its structural organization, and spinal cord injury (SCI) often results in permanent neurological deficits [184]. Current clinical treatments, such as surgical intervention, pharmacotherapy, and rehabilitation training, offer limited success in restoring neural function. In recent years, stem cell‐loaded nerve conduits have emerged as multifunctional platforms for cell and drug delivery. These conduits not only provide biophysical support but also guide cellular differentiation and neural regeneration. In particular, tubular nerve conduits have been proven to facilitate tissue infiltration and directional axonal growth.

However, most existing conduits suffer from simplistic structural designs and low regenerative efficiency, making them inadequate for addressing the complexity of SCI repair. Therefore, the development of advanced nerve conduits with hierarchical architectures and biological functionality remains a key challenge in SCI therapy. Freeze‐casting, as a strategy for constructing microscale hierarchical structures, offers significant advantages for fabricating artificial nerve conduits. Given the lack of self‐repair capability in spinal cord injuries, suitable engineered conduits are needed to bridge lesion gaps and provide a favorable microenvironment for tissue regeneration [185]. Yuan et al. [186] proposed a freeze‐casted artificial nerve conduit for SCI repair. This nerve conduit was fabricated from silk fibroin and crosslinked via alcohol fumigation [187]. The hierarchically aligned channels within the conduit allowed for the spatially defined placement of mesenchymal stem cells (MSCs) in the central region and Schwann cells (SCs) peripherally, thereby mimicking the spatiotemporal cellular distribution of native spinal cord tissue (Figure 9b). Such intricate microstructures are difficult to achieve through other methods, but dynamic ice crystal growth coupled with deliberate macroscopic design allows for the intelligent customization of required neural conduit microstructures. To evaluate the in vivo therapeutic effects, the damaged spinal cord tissue was collected 8 weeks after implantation. Macroscopic examination revealed good integration between the biomimetic nerve conduit (BNC) implant and host tissue, in contrast to the disorganized architecture seen in the non‐treated control group (Figure 9b). Hematoxylin‐eosin (HE) staining demonstrated that the lesion areas in the BNC@MSCs and BNC@MSCs/SCs groups were filled with solid tissue that was well integrated with surrounding normal tissue. In comparison, the control group exhibited large cavities and disrupted structures, while the BNC group showed significantly fewer voids. These findings suggest that the hierarchical porous structure of BNC effectively bridges SCI gaps, and the incorporation of MSCs and SCs further enhances tissue regeneration and functional recovery following spinal cord injury.

### 3.5 Acute Liver Failure

Liver injury or liver failure can result in severe metabolic disturbances, coagulopathy, and multi‐organ dysfunction, ultimately posing a serious threat to life. Major etiologies include viral infections (e.g., HBV, HCV), drug‐induced toxicity (such as acetaminophen overdose), alcohol abuse, and autoimmune diseases. Current treatments primarily rely on supportive care, liver transplantation, or artificial liver support systems. However, these approaches are often limited by donor shortages and high costs. Therefore, future therapeutic strategies are shifting toward stem cell therapy, bioengineered livers, and regenerative medicine to enable functional liver tissue repair.

**FIGURE 10:**
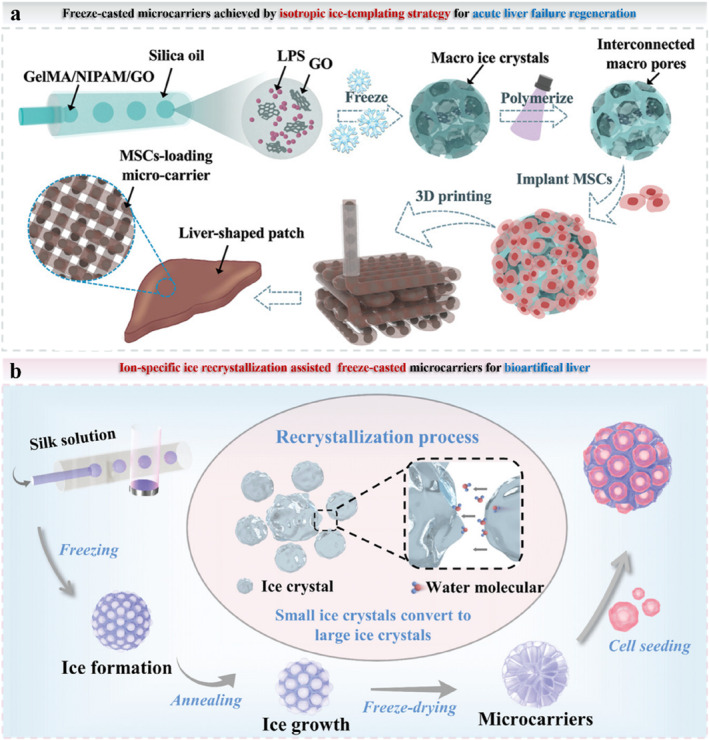
Freeze‐casted microcarriers for liver regeneration. (a) Freeze‐casted microcarriers for acute liver failure regeneration [188]. (b) Ion‐specific ice recrystallization assisted freeze‐casted microcarriers for bioartifical liver [189]. (a) Adapted with permission [188]. Copyright 2022, Wiley‐VCH. (b) Adapted with permission [189]. Copyright 2024, Wiley‐VCH.

Mesenchymal stem cell (MSC) transplantation has shown promising therapeutic effects in liver injury models. Nevertheless, traditional cell implantation often suffers from low cell viability and poor engraftment due to host immune attacks and foreign body reactions. To protect transplanted cells from immune‐mediated cytotoxicity and to enhance targeted delivery, various hydrogels and microcarriers have been explored to encapsulate MSCs. Among them, freeze‐casting technology has gained attention for its superior ice‐templating capability, which enables precise customization of microcarrier structures. Wang et al. [188] introduced an ice‐templated microcarrier system for MSC delivery to treat acute liver failure (Figure 10a). This microcarrier was composed of graphene oxide (GO), poly(N‐isopropylacrylamide) (PNIPAM), and gelatin methacrylate (GelMA). The porous structure generated through freeze‐casting allows for efficient loading of cells or drugs. Moreover, the photothermal effect of GO and the thermo‐responsibility of PNIPAM enable the microcarrier to shrink under near‐infrared (NIR) irradiation, thereby achieving controlled release of encapsulated cells or drugs. Meanwhile, the biocompatibility of GelMA facilitates cell adhesion and engraftment. Together, these functionalities make ice‐templated microcarriers highly effective for MSC delivery and significantly enhance liver regeneration.

Beyond conventional freeze‐casting, ice recrystallization‐assisted freeze‐casting, which leverages Hofmeister effect‐driven ionic regulation to control final pore sizes, has shown great potential for fabricating microcarriers tailored for liver failure treatment. For instance, Lin et al. [189] applied this strategy to construct ice‐templated microcarriers (Figure 10b) and incorporated them into a bioartificial liver system tested in rabbit models. The authors developed a high‐throughput bioreactor by integrating ice‐templated microcarriers with semi‐permeable microtubules. This system leverages ice recrystallization to regulate the formation of microstructures within the microcarriers, thereby reconstructing a three‐dimensional growth microenvironment that mimics the conditions for mature liver cells. This approach enables high‐density hepatocyte culture with enhanced biological function. This group further advances the construction of liver‐on‐a‐chip platforms and artificial liver systems. The results demonstrated significantly reduced liver enzyme levels and improved survival rates from 33% to 68%, highlighting the promising application of ice‐templated microcarrier‐integrated systems for acute liver failure therapy.

## 4 Summary and Outlook

In summary, ice‐templating has emerged as a powerful and versatile strategy for fabricating tissue engineering materials with precisely tailored architectures. By exploiting the physical and chemical ice‐templating strategies, this technique enables the construction of hierarchical, anisotropic, and interconnected porous networks that closely mimic native tissue structures. As discussed in this review, recent advances have expanded ice‐templating into a broad range of tissue engineering contexts, including cell culture scaffolds, wound healing matrices, bone regeneration constructs, nerve guidance conduits, and liver tissue engineering. These studies demonstrate that by controlling freezing parameters, additives, and material compositions, ice‐templating can modulate pore size, connectivity, and orientation, thereby influencing cell adhesion, proliferation, migration, and tissue integration. In addition, the low temperature processing conditions make it particularly suitable for incorporating bioactive molecules, living cells, and other thermosensitive components, further enhancing its potential in drug delivery, tissue engineering and regenerative medicine.

Despite these advances, several challenges must be addressed before ice‐templated materials can achieve widespread clinical translation. First, the mechanistic relationships between ice crystal growth dynamics, resulting pore architectures, and subsequent biological performance remain insufficiently understood, limiting the ability to rationally design scaffolds for specific medical needs. Second, the material scope, while diverse, still faces trade‐offs between mechanical strength, degradation rate, and bioactivity, and some functional materials exhibit poor stability or dispersion during the freezing process. Third, the scalability and reproducibility of ice‐templated scaffolds or microcarriers are hindered by sensitivity to freezing conditions, solution composition, and structure‐immobilization methods, posing difficulties for large‐scale manufacturing. Fourth, clinical translation is challenged by the lack of standardized evaluation protocols, long‐term in vivo safety data, and regulatory pathways tailored to such structurally complex biomaterials. Finally, most current studies focus on single‐function designs, whereas clinical scenarios often demand multifunctional platforms.

Several promising research directions could help unlock the full potential of ice‐templating in the tissue engineering field. A key priority is the development of multiscale quantitative models with AI assistance linking ice crystal growth, pore architecture, and biological outcomes, which would enable predictive design of scaffold structures. Expanding the material library to include novel biodegradable polymers, bioactive ceramics, and functional nanocomposites will further enhance the versatility of this approach. Integration with advanced manufacturing technologies such as 3D printing, microfluidics, and external field control (e.g., temperature gradients, magnetic or electric fields) could enable programmable and patient‐specific architectures. Furthermore, combining ice‐templating with photothermal materials enables light‐responsive smart drug release. Beyond that, leveraging continuous ice‐templating technology along with cell‐adhesion factors and signal‐sensing elements could lead to the development of cell‐sensing devices. Such devices would monitor cellular activities and biomolecular changes, playing a significant role in cell research and drug development. Multifunctional designs that couple ice‐templated structures with drug release systems, biosensing components, and external stimulus responsiveness could create next‐generation therapeutic platforms. Moreover, as shown in Table 4, a number of ice‐templated microporous biomaterials have already entered the market. Since the pioneering work of Yannas and Burke [201], the process maturity and biological efficacy of this established technique have been thoroughly validated. The commercial success of products like the Integra Dermal Regeneration Template is currently founded on conventional freeze‐drying‐based ice‐templating technology [190, 191]. In contrast, other emerging ice‐templating techniques, such as those capable of fabricating anisotropic pore structures, have not yet progressed to the clinical stage, despite demonstrating superior potential for tuning physicochemical properties and attracting significant attention in fundamental research. In future, as the understanding of the structure–property relationships in these advanced materials deepens, such emerging technologies hold great promise for developing the next generation of high‐performance implants, with broad prospects for future clinical translation. Robust preclinical studies, standardized performance metrics, and scalable and cost‐effective fabrication methods will be essential to bridge the gap from laboratory innovation to clinical practice.

**TABLE 4:** Examples of clinically used ice‐templated microporous biomaterials fabricated by freeze‐drying strategy.

Material	Representative product	Primary application areas	References
Collagen and chondroitin‐6‐sulfate (CS)	Integra wound reconstruction and care range of products (integra LifeSciences)	Extensive burns Burn scar reconstruction Diabetic foot ulcer	[190, 191]
Collagen and elastin hydrolysate	MatriDerm (MedSkin solution Dr. Suwelack AG)	Full thickness skin defects	[192, 193]
Collagen and rhBMP‐2	InFUSE bone graft (Medtronic)	Anterior lumbar spine fusion Tibial trauma Oral maxillofacial reconstruction	[194, 195]
Cellulose and collagen	PROMOGRAN protease modulating matrix (Acelity L.P. Inc. KCI)	Diabetic, pressure, venous ulcers Traumatic and surgical wounds	[196, 197]
Collagen	DuraGen (integra LifeSciences)	Dura mater repair	[198]
Human decellularized dermis	AlloDerm (Lifecell), AlloMax (CR Bard/Davol Inc.), DermaMatrix acellular dermis (synthes)	Skin defects Soft tissue repair Hernia repair Breast reconstruction	[199, 200]

Overall, ice‐templating provides a unique and adaptable platform for engineering biomedical materials with finely tuned structural and functional properties. Continued interdisciplinary collaboration among materials scientists, bioengineers, and clinicians will be critical for overcome existing challenges and accelerate the translation of ice‐templated constructs into impactful clinical solutions.

## Author Contributions

Shuangshuang Miao, Wanchuan Ding, and Jingjing Gan conceived the idea and the proposal. Shuangshuang Miao wrote the manuscript. Xingkui Guo, Chenhui Bai, Wei Zhou, and Yiwen Wu revised the manuscript. Shuangshuang Miao prepared figures with inputs from Xingkui Guo and Jingjing Gan. All authors discussed and commented on the manuscript.

## Ethics Statement

This review is only a summary of the existing literature and does not refer to any human or animal experiments.

## Consent

The authors have nothing to report.

## Conflicts of Interest

The authors declare no conflicts of interest.

## Data Availability

Data sharing not applicable to this article as no datasets were generated or analyzed during the current study.
